# Selection by Pollinators on Floral Traits in Generalized *Trollius ranunculoides* (Ranunculaceae) along Altitudinal Gradients

**DOI:** 10.1371/journal.pone.0118299

**Published:** 2015-02-18

**Authors:** Zhi-Gang Zhao, Yi-Ke Wang

**Affiliations:** State Key Laboratory of Grassland and Agro-ecosystems, School of Life Sciences, Lanzhou University, Lanzhou, 730000, P.R. China; Indian Institute of Science, INDIA

## Abstract

Abundance and visitation of pollinator assemblages tend to decrease with altitude, leading to an increase in pollen limitation. Thus increased competition for pollinators may generate stronger selection on attractive traits of flowers at high elevations and cause floral adaptive evolution. Few studies have related geographically variable selection from pollinators and intraspecific floral differentiation. We investigated the variation of *Trollius ranunculoides* flowers and its pollinators along an altitudinal gradient on the eastern Qinghai-Tibet Plateau, and measured phenotypic selection by pollinators on floral traits across populations. The results showed significant decline of visitation rate of bees along altitudinal gradients, while flies was unchanged. When fitness is estimated by the visitation rate rather than the seed number per plant, phenotypic selection on the sepal length and width shows a significant correlation between the selection strength and the altitude, with stronger selection at higher altitudes. However, significant decreases in the sepal length and width of *T. ranunculoides* along the altitudinal gradient did not correspond to stronger selection of pollinators. In contrast to the pollinator visitation, mean annual precipitation negatively affected the sepal length and width, and contributed more to geographical variation in measured floral traits than the visitation rate of pollinators. Therefore, the sepal size may have been influenced by conflicting selection pressures from biotic and abiotic selective agents. This study supports the hypothesis that lower pollinator availability at high altitude can intensify selection on flower attractive traits, but abiotic selection is preventing a response to selection from pollinators.

## Introduction

Plant-pollinator interactions are among the most active subjects of evolutionary biology since Darwin[1–3]. Animal pollinators are believed to have played a key role in the diversification of angiosperm flowers [1–5]. The intensity of plant-pollinator interactions are frequently correlated with geographic variation of flower traits in a clinal or mosaic fashion and shows environment-specific selection by pollinators [6–9], although abiotic factors can also account for geographical variation of flowers under stressful conditions [10–11]. Based on the most effective pollinator principle [2], floral traits can evolve to correspond to the morphology, physiology and behavior of specific pollinators [12–13]. Pollinator assemblages often vary geographically in types and abundances of insects [14–20]. Thus, the geographical variation in floral morphology seen in many plant species may depend on differences in the species composition of pollinator assemblage, as well as visitation frequency and pollination efficiency [9, 13, 21–23]. Consequent geographical variation of selection mediated by pollinators can produce clinal variation in flower size [24–25]. However, few studies have addressed selection on floral traits across populations with different pollinator assemblages [26–28], and rarely related intraspecific floral differentiation to geographically changing selection from pollinators.

Alpine environments are an ideal ecological context to explore adaptation of plants along altitudinal gradients [29]. Abundance and visitation of pollinator assemblages tend to decrease with increasing altitude, leading to an increase in pollination limitation [30–33]. Thus, increased competition for pollinators is likely to generate stronger selection on attractive traits of flowers at high elevations. This may generate clinal variation in selection regimes and consequently cause the evolutionary divergence of floral traits along altitudinal gradients [34–36]. Plants may adapt to lower pollinator visitation at higher altitudes by enhancing attractiveness or increasing floral longevity when competition for limited pollinator service is intensified [32, 36]. It has been shown that high-altitude plants allocate proportionally more of their aboveground biomass to floral structures rather than leaves compared to lowland plants [37]. Although plant size is generally smaller in high altitude populations, flower size in many alpine plant species also increases with altitude, and such clinal variation in floral traits has often been explained as corresponding to local pollinator assemblages or visitation frequencies [11, 34–36, 38–40]. A significant correlation between floral size and elevation is predicted by phenotypic selection exerted by pollinators [24, 25]. However, geographic variation in flower traits could also be associated with climatic factors [10–11]. Because local climatic conditions (e.g. precipitation and temperature) usually vary with altitudes, adaptive variation of floral size in responses to pollinator-mediated selection may conflict with selection from the abiotic environment [41]. Furthermore, a full understanding of selection on floral traits requires determining the relative contributions of abiotic and biotic factors in the geographical differentiation of floral traits [4, 23, 27, 42].

The present study investigates the clinal variation in *Trollius ranunculoides* flowers and its pollinators along an altitudinal gradient on the eastern Qinghai-Tibet Plateau, and examines population-level phenotypic selection by pollinators. *T*. *ranunculoides* is a self-incompatible hermaphrodite with a generalized pollination system [43], covering an extensive range of altitude in alpine meadows at Qinghai-Tibet Plateau. Flowers of *T*. *ranunculoides* vary between alpine and subalpine populations, corresponding to a change of pollinator assemblages with reduced bee visitations but increased visitation from flies at higher altitudes [20]. If the differences in pollinator communities previously shown in discrete populations hold along the entire altitudinal gradient, we predict that lower visitation frequency of pollinators in high altitude would result in stronger selection by pollinators on larger flowers of *T*. *ranunculoides* due to stronger competition for pollination service. We focus specifically on phenotypic selection that occurs through visitation of pollinators among populations [44–45]. In addition, we examine associations of climatic factors (mean temperature and precipitation) with floral traits and visitation rate of pollinators, to determine the relative importance of pollinators and abiotic factors to floral variation in *T*. *ranunculoides*. We predict that floral variation is driven by altitudinal variation in pollinator visitation frequency.

## Materials and Methods

### Ethics Statement

No permits were required to carry out this study. The owner of all the study sites is P.R China. The Chinese government gives us permission to conduct the study on these sites. We confirm that the field studies did not involve endangered or protected species. No vertebrate were studied for this manuscript.

### Study species

**Fig 1:**
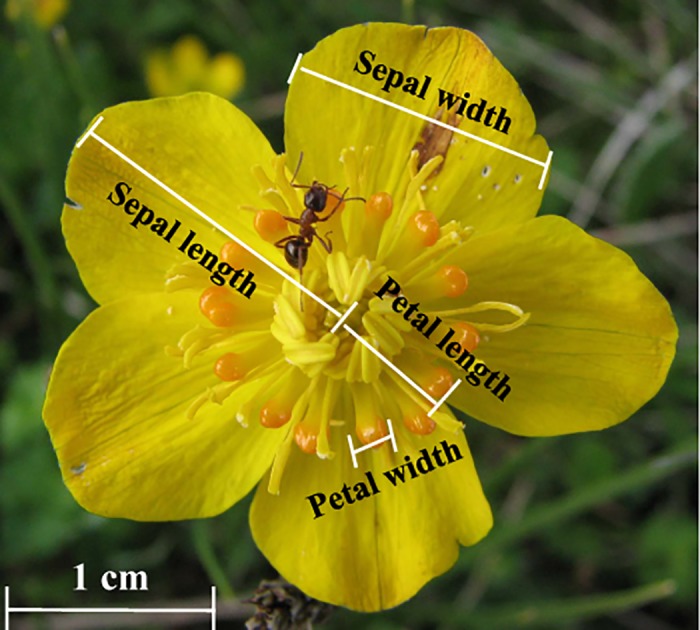
*Trollius ranunculoide* flower with measured traits.

*Trollius ranunculoides* Hemsl. (Ranunculaceae) is a hermaphroditic perennial in alpine meadows (from 2900 to 4100 m a.s.l.) in east Qinghai-Tibet Plateau. It usually occurs along small streams and wet meadows or under shrubs. The plant generally produces a single bright yellow flower on an erect stalk 6–20 cm high, while a few individuals produce two or three flowering stalks. The bowl-shaped flower consists of 5 yellow petal-like sepals and several stamen-like golden yellow petals (Fig. 1). The stamen-like petals are shorter than stamens and have an expanded terminus. A nectary is located at the base of each petal. Flowering begins from early June to mid-July, and one flower generally lasts ∼8 d. *T*. *ranunculoides* is self-incompatible, and seed production depends on insect pollinators composed of bees, flies, ants and occasionally beetles [43].

### Pollinator Observations and Flower Measures

**Fig 2:**
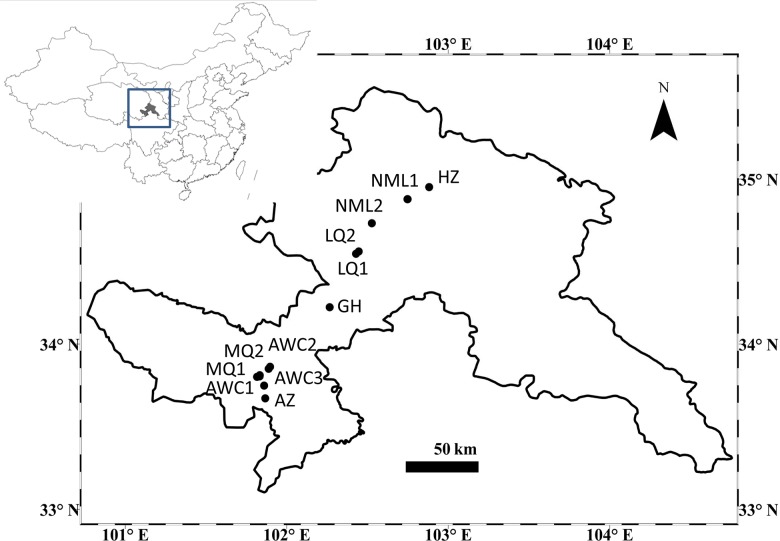
Location of the 12 studied populations of *T*. *ranunculoides* along an altitudinal gradient of southwest Gansu Province on the eastern Qinghai-Tibet Plateau. The map was generated in ArcGIS 9.0 (ESRI, Environmental Systems Research Institute, Inc.).

In the 2010 flowering season, we surveyed 12 populations (33°40′N-34°57′N; 101°48′E-102°52′E) of *T*. *ranunculoides* along altitudinal gradients of southwest Gansu Province on eastern Qinghai-Tibet Plateau (Fig. 2, S1 Table). The selected populations were representative of the altitudinal gradient in the area. In the middle of the flowering season in each population, six observers recorded flower visitors to determine the composition, relative abundance and visitation frequency of the pollinator assemblages between 8:30 a.m.-17:30 p.m. on sunny days. Fifty to sixty individuals that were at least 0.5 m apart and with at least half of the anthers dehisced were haphazardly tagged in each population. Each observer simultaneously watched three plants within the range of vision for a 15-min observation period in each population. On average, five observations were conducted for each plant in each population per day on two successive sampling days. Six observers started at the same time and shifted to other three plants once finished, so each plant can be evenly observed in turn by all observers. During the observations, the observer approached focal plants closely enough (generally 1–2 m away) to identify the smaller visitors and capture for identification, without disturbing visits to the flowers. The visiting insects included flies, hoverflies, solitary bees, honey bees, ants, beetles and beeflies (S2 Table). The insects visiting *T*. *ranunculoides* flowers can be grouped into two main functional groups of bees and flies based on insect visiting behavior, habits and frequency [20, 46]. Ants were often observed in the higher populations, but they always collected nectar and rarely contacted reproductive parts of the flower and thus were excluded in later analyses. From the visitation data of pollinators (all insects except for ants), we computed visitation frequency (i.e. no. of visits plant^-1^ h^-1^). A total of 200 observation periods totaling 50 h were completed in each of 12 populations.

After observations were finished in each population, we measured flower stalk height (FH), sepal length and width (SL and SW), and petal length and width (PL and PW) (Fig. 1) with digital calipers (Mahr Federal 16 ER Digital Caliper, Germany) precise to 0.01 mm. If an individual produced more than one flower, all flowers were measured and averaged. We tried to collect seeds from the plants used for pollinator observation at the end of the season of 12 populations. Due to destruction of plants and identifying tags by livestock in all sampling populations, fruit collection was severely limited.

Differences among populations in floral traits, visitation rates of the two functional groups, and visitation rates of all insects pooled were tested using one-way ANOVAs. Altitudinal trends in floral traits were tested by regressing individual plant values on altitude. We also checked within- and among-population relationships between pollinator visitation and seed set by regression analyses, although this study focused on pollinator visitation.

### Effects of Biotic and Abiotic Factors

To determine effects of climatic factors on floral traits and visitation of pollinators, we estimated environmental factors (e.g. mean temperature and precipitation) for each population using information available from WorldClim database (http://www.worldclim.org/) [47]. Bioclimatic variables were obtained for each site at a spatial resolution of 1 km^2^. From the available information we selected annual temperature and precipitation averages (S1 Table), which correspond to average values obtained from interpolations of data observed during the period between the years 1950 and 2000.

The relationships between environmental factors, floral traits and pollinator visitation were determined by simple regressions. To analyze relative contribution of biotic (visitation rate of pollinator assemblages) and abiotic (climatic) factors to the variation of floral traits, a redundancy analysis (RDA) was performed using the package biodiversityR of the R 2.15.2 statistical software (R Development Core Team, 2010). For this analysis, a matrix of population × floral traits was analyzed in relation to a corresponding matrix of explanatory variables. We included four explanatory factors that were hypothesized to be important determinants of variation in flower traits: mean annual temperature, mean annual precipitation, visitation rates of bees and flies. To avoid using different scales and for comparative purposes, the variables were standardized to zero mean and unity standard deviation. The significance of the variability explained by each environmental factor was analyzed by automatic selection of variables using a Monte Carlo test with 999 permutations. In this procedure, the variable that best fits the data is selected first and then the next best fitting variable is added to the model.

### Phenotypic selection on floral traits

To estimate the magnitude and direction of selection acting on the floral traits (flower height, sepal length and width, petal length and width), we conducted phenotypic selection analysis in univariate and multivariate models for each population [48]. We used the visitation rate of all pollinators per plant as a fitness estimate, and relativized fitness by dividing by mean fitness. We then standardized each trait to mean 0 and variance 1 to facilitate comparison among traits and between populations. We first estimated the selection differentials (*S*) by regressing the relative visitation rate on a standardized single trait which described selection for each floral trait including selection acting directly on the trait and selection acting on correlated traits [48–49]. We also calculated the standardized linear selection gradients (β) by regressing relative visitation rate on the standardized traits simultaneously, in a multiple regression. Standardized selection gradients can describe the direct selection on a trait, after accounting for selection on the other traits measured. Similarly, we also estimated selection differentials and selection gradients using seed number per plant as fitness component in 8 of 12 populations excluding four populations in which individuals with intact fruits were less than 30. We used principal component analysis (PCA) with varimax rotation to reduce the dimensionality due to highly correlated between all floral traits. This can result in a reduced set of composite variables (PCs) which retain the maximum total variation on uncorrelated axes [15, 24, 48]. The first four principal components accounted for more than 95% of the total trait variance. Thus the selection gradient analysis was conducted on the first four PCs from the PCA as independent variables. PCA showed that the sepal length and width loaded heavily on the PC1, the petal length on PC2, the petal width on PC3 and the flower height on PC4 (S3 Table). Because the visitation rate in some populations was non-normally distributed, for those populations we used square root-transformed visitation rate to meet the assumption of normality in estimating selection differentials and gradients. However, transformed relative fitness is no longer an unbiased estimate [48]. Therefore, when the transformation was necessary, we reported the selection differential or gradient from the untransformed model, but the P-value from the transformed model [50].

The variation in selection differentials and selection gradients with altitude was analyzed by a linear regression. The correlations between selection differentials and population means of measured floral traits were also analyzed to examine whether mean floral traits were related to the magnitude of selection at population level. We also repeated above phenotypic selection analyses by using the visitation rates of bees and flies respectively as a fitness estimate. All statistical analyses were performed in JMP 7.0 (SAS Institute 2007).

## Results

### Clinal variation in floral traits, seed set and visitation rate of pollinator assemblages

**Fig 3:**
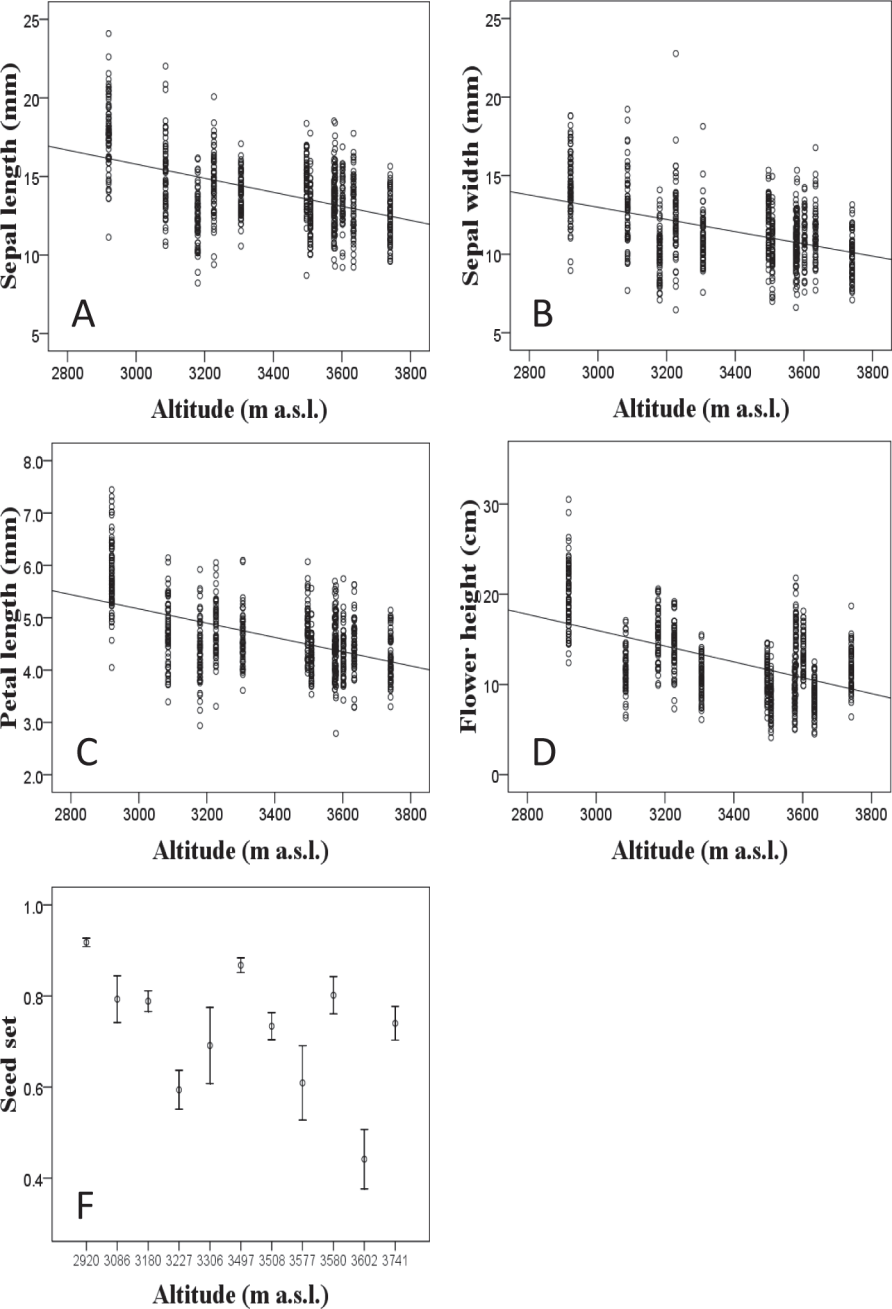
Clinal variation in the floral traits and seed set of *T*. *ranunculoides* populations located along the altitudinal gradient of Gansu Province on the eastern Qinghai-Tibet Plateau.

**Fig 4:**
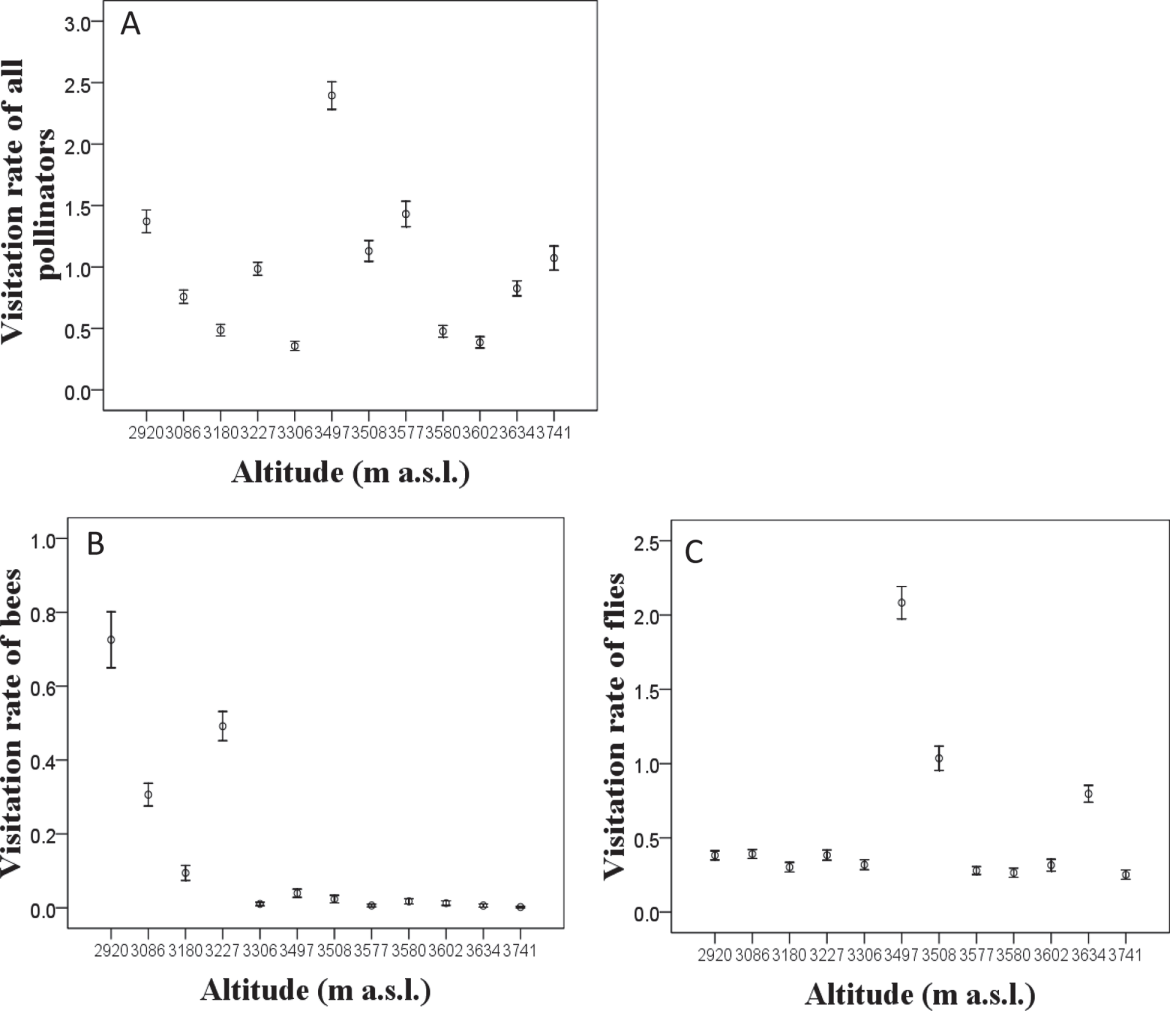
Clinal variation of the visitation rate (visits plant^-1^ h^-1^) of all pollinators and main assemblages (flies and bees) across *T*. *ranunculoides* populations located along the altitudinal gradient.

The floral traits, the seed set and the visitation rates of total pollinators and two main pollinator assemblages (bees and flies) varied significantly among populations of *T*. *ranunculoides* (Table 1). Except for petal width, the floral traits (the sepal length and width, the petal length and the flower height) decreased significantly with altitude; the mean seed set per plant was also relatively lower at high altitude (Fig. 3). The visitation rate of total pollinators decreased along the altitudinal gradient (Fig. 4, *r* = -0.206, *P*<0.001). Whereas the visitation rate of bees decreased significantly from low- to high- altitude (*r* = -0.663, *P* = 0.019), the visitation rate of flies was relatively constant except for three populations which received higher visitation by flies (Fig. 4). All floral traits (except floral height and petal width) were positively correlated (Table 2).

**Table 1:** ANOVAs to test the variation of plant traits and visitation frequency of pollinators among populations of *T*. *ranunculoides*.

Source	df	SS	*F*	*P*
Sepal length	11	1663.69	45.9	<0.001
Sepal width	11	1222.1	34.7	<0.001
Petal length	11	141.2	46.7	<0.001
Petal width	11	6.1	22.3	<0.001
Flower height	11	8432.3	129.2	<0.001
Seed set	10	8.72	14.8	<0.001
Visit of all pollinators	11	211.8	55.3	<0.001
Visit of bees	11	41.8	64.3	<0.001
Visit of flies	11	185.3	104.9	<0.001

**Table 2:** Pearson’s correlation coefficients (*r*) for the floral traits measured in 12 populations of *T*. *ranunculoides*.

Trait	Sepal width	Petal length	Petal width	Flower height
Sepal length	0.76 ***	0.73 ***	0.35 ***	0.49 ***
Sepal width		0.67 ***	0.40 ***	0.41 ***
Petal length			0.29 ***	0.46 ***
Petal width				0.008 ns

Number of individuals of all population, *N* = 711.

*** *P*< 0.0001; ns, nonsignificant

### The relation of floral traits with biotic and abiotic factors

Along the altitude gradient, the visitation rate of bees rather than that of flies and total pollinator was positively correlated to the sepal length and width across populations (both *r* >0.7, *P*<0.01). Mean annual precipitation was negatively correlated with the sepal length and width, flower height, and the visitation rate of bees (all *r* < -0.6, *P*<0.05); by contrast, these floral traits and the bee visits increased significantly with mean annual temperature (all *r* > 0.6, *P*<0.05). The RDA that analyzed the association between floral traits and environmental variables showed that the mean annual precipitation (*F* = 7.59, *P* = 0.009) and the visitation rate of bees (*F* = 3.98, *P* = 0.064) contributed significantly to the model. However, the mean annual precipitation obviously contributed more to the variation of floral traits, countering selection from bees (S1 Fig).

### Phenotypic selection analysis along the altitudinal gradient

**Fig 5:**
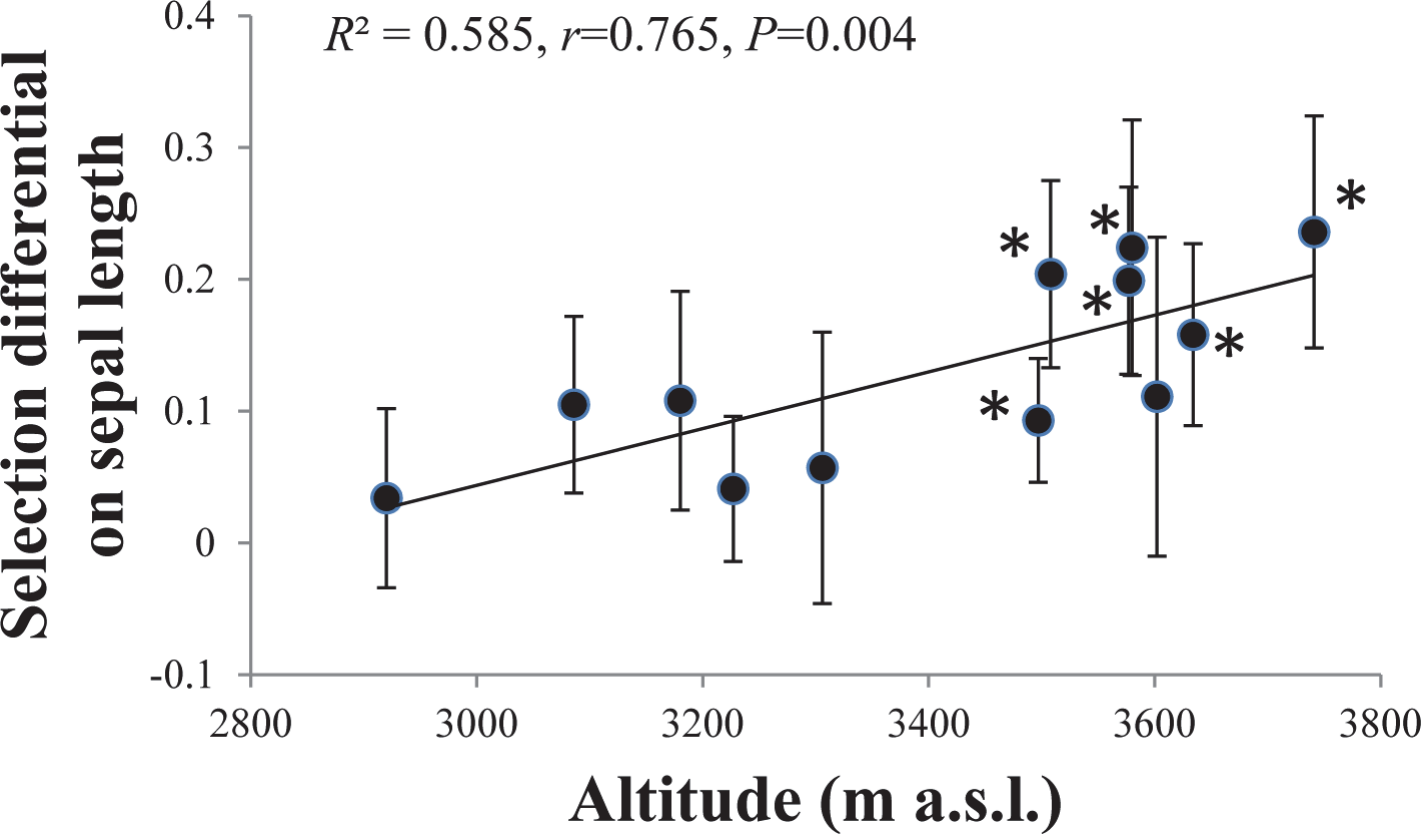
Selection differentials on sepal length *T*. *ranunculoides* along the altitudinal gradient. Mean selection differential ± SE, each point represents a population. * indicates statistical significance.

Significant selection differentials estimated by the visitation rate were more frequent on the sepal length and width than other traits, and were found in more than six populations (S4 Table). Linear selection differentials on sepal length increased along the altitudinal gradient (Fig. 5), but no altitudinal trend was found for the selection differentials on sepal width. The strongest selection differential on sepal length occurred in the population with the smallest mean sepal length as showed by a negative correlation between the mean sepal length and the selection differential (S2 Fig). By contrast, selection differentials estimated by the seed number per plant were less significant in studied populations (S4 Table), without obvious trend with the altitudinal gradient.

**Fig 6:**
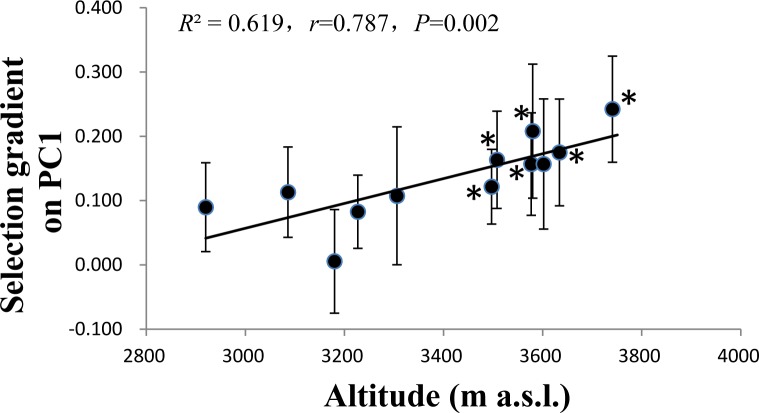
Selection gradients on PC1 along the altitudinal gradient. Mean selection gradient ± SE, each point indicates a population. PC1 represents the sepal length and width, which was from principal component analysis with varimax rotation. * indicates statistical significance.

Significant linear selection gradients estimated by the visitation rate were more frequently on the PC1 rather than other PCs, and were found in half of the populations (S5 Table). As with the selection differentials, the magnitude of selection gradients on the PC1 increased significantly along the altitudinal gradient (Fig. 6), showing stronger directional selection by pollinators on the sepal length and width in high-altitude populations of *T*. *ranunculoides*. However, selection gradients estimated by the seed number per plant were still less significant in studied populations (S5 Table).

We also repeated phenotypic selection analyses by using the visitation rates of bees and flies respectively as a fitness estimate, but the results had no significant trends and thus were not given.

### Relationship between visitation rate of pollinators and seed set

At population level, marginally significant relationship between the mean visitation rate of pollinators and the mean seed set showed that high pollinator visitation can lead to increased seed set in *T*. *ranunculoides* (S3 Fig). Within populations, significant correlations were only found in three of all populations (S6 Table), suggesting population-specific pollination limitation.

## Discussion

Previous studies in mountain environments show that decreases in the abundance and visitation rate of pollinator assemblages that correspond with increasing altitude increase pollination limitation [32]. For *T*. *ranunculoides* in the Qinghai-Tibet Plateau, the total visitation of pollinators decreased with altitude, with the visitation rate of bees decreasing significantly with altitude and fly visitation staying constant. In a previous study, we found that seed set in *T*. *ranunculoides* was pollen limited in a high-altitude population compared to a lower population [51]. Although a supplemental pollination experiment was not conducted in this study, there was a positive relationship between the visitation rate of pollinators and seed set, which was marginally significant at the population level. This suggests a trend that low visitation rate of effective pollinators will reduce mean seed set of *T*. *ranunculoides* populations along an altitudinal gradient. Within populations, however, significant correlation between pollinator visitation and seed set occurred only in three populations, and was especially unusual in higher populations (over 3500m). It shows that seed set at high altitudes may be constrained by abiotic environments, not by pollen availability alone [52–53]. An association between reduced pollination service and increased pollen limitation also existed at the margin of geographic range in an annual plant [54]. The correlation between variation in reproductive success with pollinator availability has been addressed in other studies [55].

Low pollinator visitation may increase competition among individual plants for pollinator service and consequently produce stronger positive selection on floral traits that attract pollinators. Two floral traits of *T*. *ranunculoides*, the sepal length and width, were under significant selection by pollinators, and the magnitude of selection estimated by the visitation rate of pollinators significantly increased with altitudes as presented by significant correlations between the selection differentials and the selection gradients with the altitude. This strongly indicates more intense selection on floral size of *T*. *ranunculoides* at high altitude where the visitation frequency of pollinators was lower. This is the first study to demonstrate that pollinator-mediated selection on floral traits increased with altitude as a result of decreased pollinator visitation. However, floral traits studied in *T*. *ranunculoides* except for the petal width decreased significantly with altitude, especially the sepal length and width, and have not increased in response to such stronger selection by pollinators along the altitudinal gradient. This observation is inconsistent with our expectation. The results, in combination with the finding of the strongest selection differential occurred in the population with the smallest sepal length, show that the sepal length is under selection from pollinators but has not evolved accordingly. One possible cause is that current populations of *T*. *ranunculoides* are not at their adaptive optima, which is likely caused by recent colonization. Another possibility is that the selection of pollinators is currently counteracted by other selection pressures (e.g. abiotic factors) which constrain the evolution of sepal length. It should be noted that the selection strength estimated by visitation rate should be limited to some extent because of its variability with the flowering season, which can be influenced by weather conditions or changes in pollinator fauna.

Increases in flower size with altitude have been documented for other alpine bee-pollinated species [11, 34–36], which suggest that the observed clinal variation is likely associated with the role of pollinators. Due to an association of flower size and pollination success within populations, increased flower size in *Cytisus scoparius* with altitude could be a result of selection by insect pollinators with a parallel increase in their body sizes along an altitude gradient [35]. Similarly, Madd *et al*. [40] also found increased flower size of *Campanula rotundifolia* with altitude; this clinal variation in flower size was attributed to different selection regimes mediated by pollinators based on lower visitation rates as well as increased bumble-bee size over altitude. By contrast, Anderson & Johnson [42] found an altitudinal cline variation in proboscis length of the flies that pollinated *Zaluzianskya microsiphon* but not in the corolla length of the flowers. However, none of these studies have estimated inter-population variation of phenotypic selection mediated by pollinators and directly demonstrated the selective role of pollinators responsible for the clinal variation in floral size with altitude. In the alpine herb *Ranunculus acris*, Totland [56] found that flowers were smaller in high altitude than in low populations. Fly visitation rates were higher in the low altitude populations relative to the high populations, with no difference in pollinator assemblages between populations. But pollen limitation at high-altitude populations of *R*. *acris* was lower than lower populations due to limitation of low temperature on seed production at high population. Consequently, more intense phenotypic selection on flower size at the low-altitude corresponded to flower size variation of *R*. *acris* between high and low populations. Nattero *et al*. [57] found that clinal variation in corolla length of *Nicotiana glauca* with altitude was associated with bill length variation of pollinators. Because corolla length was a target of hummingbird-mediated phenotypic selection, the match between the direction of selection and among-population variation of corolla length indicates intraspecific geographic differentiation in *Nicotiana glauca* flowers caused by pollinator-mediated selection [58].

Although clearly pollinators exert strong selection, evolutionary responses of floral size may also be influenced by selection from the abiotic factors [41]. In this study, selection by pollinators intensified with altitude, but floral size (the sepal length and width) of *T*. *ranunculoides* did not shift as we expected. When the effect of climatic factors was considered in studied populations of *T*. *ranunculoides*, we found that mean annual precipitation was negatively related to the sepal size and contributed more to flower variation than pollinator visitation, showing a stronger role for abiotic factors in shaping floral attractive traits of 12 *T*. *ranunculoides* populations. Furthermore, the geographic patterns of phenotypic selection estimated by the visitation rate were inconsistent with that estimated by the seed number per plant, suggesting a compromise between pollinator preferences and resource constrains may result in the clinal variation of flower size of *T*. *ranunculoides* with altitude. Floral size, unlike mechanical-fit-related traits in pollination-specialized species may be less critical in adaptive differentiation, and has often been affected by the abiotic environment [42, 59–61]. The geographical relationship between flower size and precipitation has also been reported in other studies [11, 61–62]. In *Calceolaria polyrhiza*, geographical variation of corolla size was decoupled from variation in the mechanical-fit-related traits, and was mainly affected by climatic gradients just like the vegetative traits [61]. Even in the presence of pollinator-mediated selection for larger flowers, smaller flowers may be favored by the resource-cost compromise in harsh environments [11, 41, 56, 59, 63]. Similarly, resource availability might constrain floral response to directional selection in *T*. *ranunculoides* as smaller plant sizes at high altitude would be physiologically difficult to support large flowers. However, in a previous study, we found higher biomass allocation to the sepals of *T*. *ranunculoides* at high altitude for alleviating increased pollen limitation [64]. This suggests that increased allocation to the sepals in *T*. *ranunculoides* might be selected to enhance attraction for pollinators in high-altitude populations. This is also evinced by proportionally more allocation of biomass to floral attractiveness in smaller high-altitude species than lowland species, whereas biomass allocation to leaves did not change, indicating the importance of pollinator attraction for alpine plants [37]. Flower biomass varies faster than linear floral size (e.g. length) due to an allometric relationship between them [63]. Therefore, even a small increase in flower size will incur disproportionately higher costs in terms of biomass [65]. Smaller flowers but higher allocation to sepals in *T*. *ranunculoides* can allow an improved use of reproductive resources under stressful conditions. The clinal variation of flower size in *T*. *ranunculoides* with altitude may reflect a compromise between pollinator preferences and constraints due to limited resources.

## Conclusions

We demonstrate that visitation rate of pollinators decreased with altitude, and the strength of pollinator selection on floral size of *T*. *ranunculoides* increased correspondingly along an altitudinal altitude due to intense competition for pollination service. However, reduced flower size with altitude was not consistent with intensified pollinator selection, which may be influenced by conflicting selection pressures from abiotic environments (e.g. precipitation). Our results support the hypothesis that lower pollinator availability at high altitude can intensify selection on flower attractive traits, but floral adaptive evolution may be limited by other abiotic factors at the same time.

## Supporting Information

S1 Fig Biplot of the first two axes of the RDA ordination for 12 *T*. *ranunculoides* populations (gray circles). The explanatory environmental factors (arrows and blue words with gray context) were significant (*P*<0.05) determinants of floral traits (black diamonds and red italic abbreviations). The eigenvalue associated with each axis is provided in parentheses. The explanatory variables are described in Materials and Methods; their values are reported in S1 Table.
(TIF)

S2 Fig The relationship of the sepal length and corresponding selection differential at population level of *T*. *ranunculoides*. (TIF)

S3 Fig The relationship between the visitation rate of pollinators and seed set among 9 populations of *T*. *ranunculoides*. (TIF)

S1 Table Mean annual temperature and precipitation at 12 populations along the altitudinal gradient of *T*. *ranunculoides*. (DOCX)

S2 Table Number of observed visitors by functional groups and major species in 12 populations of *T*. *ranunculoides* along an altitudinal gradient. (DOCX)

S3 Table Loadings of floral traits of *T*. *ranunculoides* on the first four components (PCs) produced by a principle components analysis with a varimax rotation. (DOCX)

S4 Table Selection differential (*S*), with fitness estimated as the visitation rate of all pollinators on the floral traits of 12 *T*. *ranunculoides* populations. (DOCX)

S5 Table Selection gradients (β), with fitness estimated as the visitation rate of all pollinators on the loadings of these traits on the first four components (PCs) produced by a principal components analysis with a varimax rotation of 12 *T*. *ranunculoides* populations. (DOCX)

S6 Table The relationships between the visitation rate of pollinators and the seed set in 11 populations of *T*. *ranunculoides*. (DOCX)
