# Supplementary figures and images for: Selection by Pollinators on Floral Traits in Generalized Trollius ranunculoides (Ranunculaceae) along Altitudinal Gradients
Source: PLoS One. 2015 Feb 18;10(2):e0118299. doi: 10.1371/journal.pone.0118299 (PMC4334720; doi:10.1371/journal.pone.0118299)

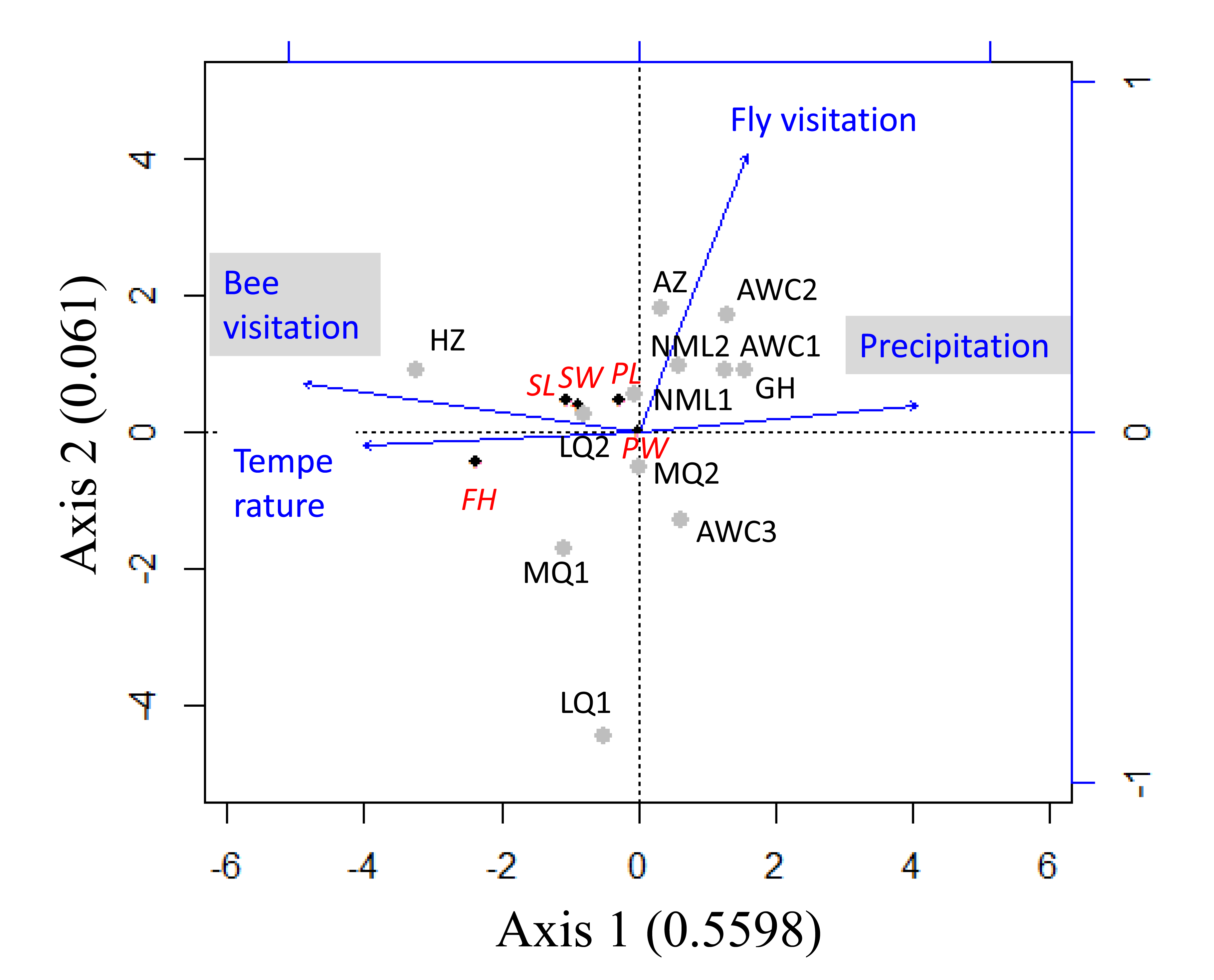

Supplement: S1 Fig — The explanatory environmental factors (arrows and blue words with gray context) were significant (P<0.05) determinants of floral traits (black diamonds and red italic abbreviations). The eigenvalue associated with each axis is provided in parentheses. The explanatory variables are described in Materials and Methods; their values are reported in S1 Table. (TIF) [file pone.0118299.s001.tif]

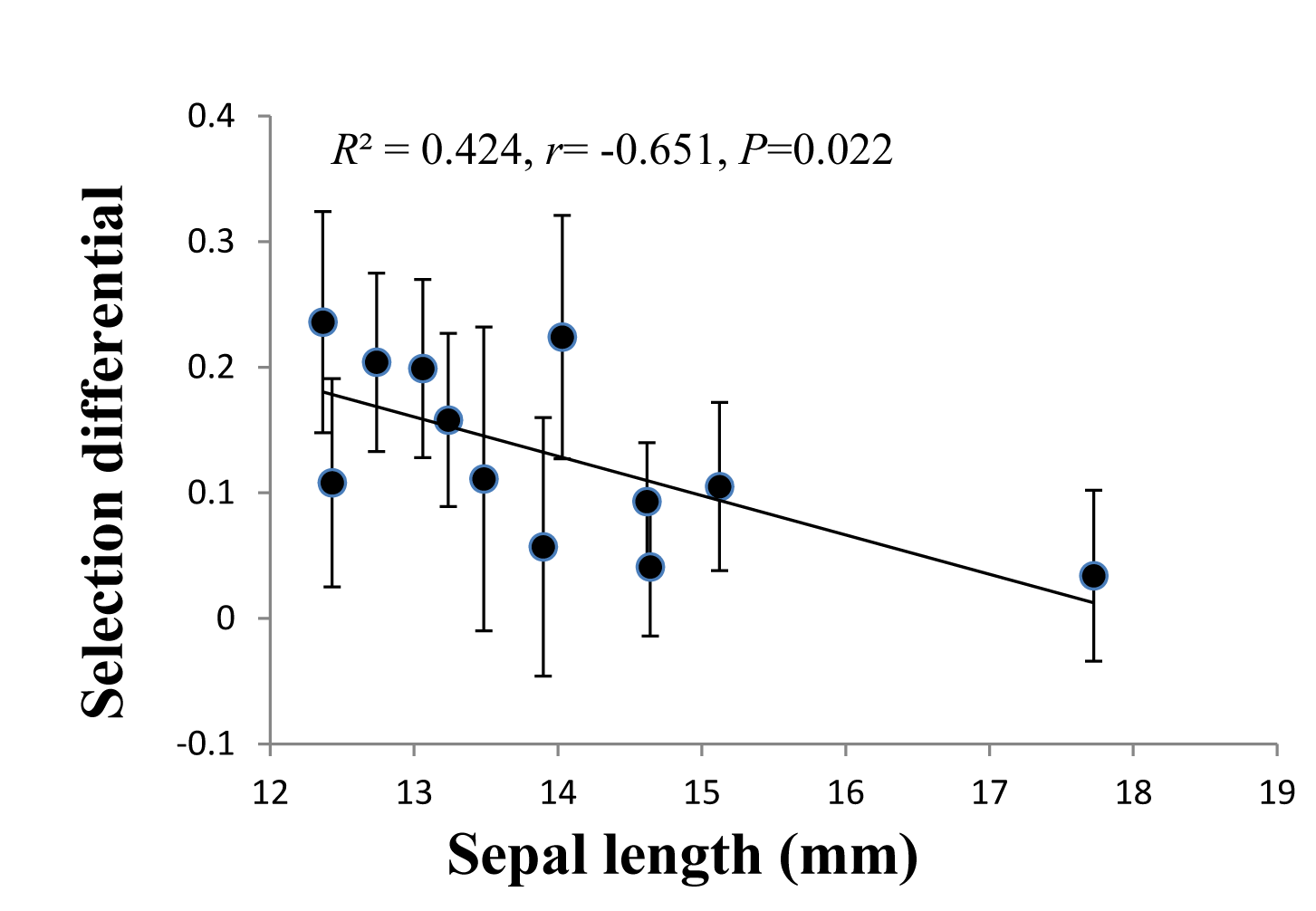

Supplement: S2 Fig — (TIF) [file pone.0118299.s002.tif]

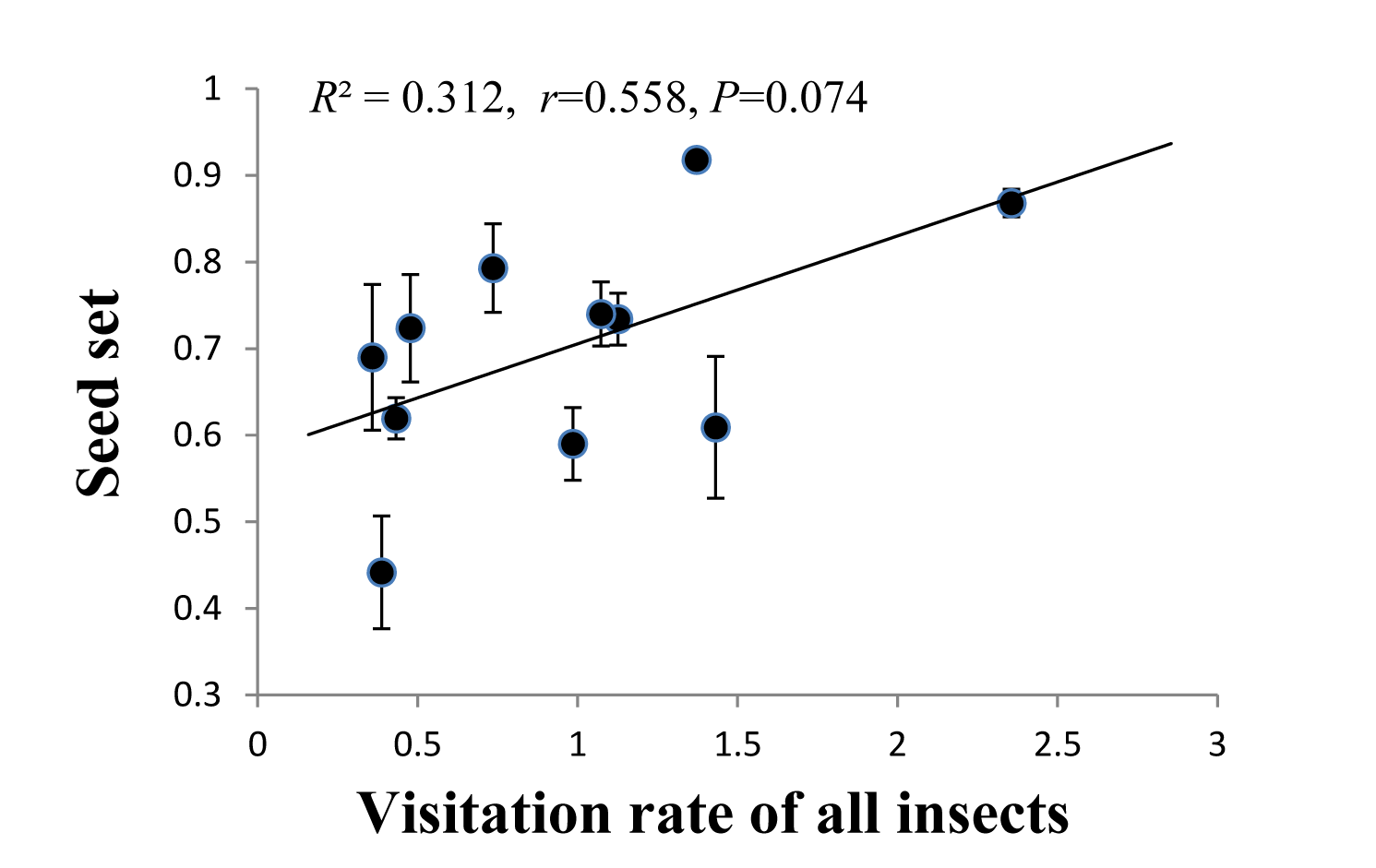

Supplement: S3 Fig — (TIF) [file pone.0118299.s003.tif]
