# Supplementary material for: Selection by Pollinators on Floral Traits in Generalized Trollius ranunculoides (Ranunculaceae) along Altitudinal Gradients
Source: PLoS One. 2015 Feb 18;10(2):e0118299. doi: 10.1371/journal.pone.0118299 (PMC4334720; doi:10.1371/journal.pone.0118299)
Supplement: S1 Table — (DOCX) [file pone.0118299.s004.docx]

**Table S1. Mean annual temperature and precipitation at 12 populations along the altitudinal gradient of *T. ranunculoides.***

| Population | Altitude (m) | Coordinates | | Mean Temperature (℃) | Mean Precipitation (mm) |
| --- | --- | --- | --- | --- | --- |
| HZ | 2920 | 34°57′02″ N | 102°52′56″ E | -0.88 | 46.92 |
| NML1 | 3086 | 34°49′55″ N | 102°39′31″ E | -2.73 | 48.08 |
| LQ1 | 3180 | 34°33′39″ N | 102°26′44″ E | -10.09 | 50.33 |
| LQ2 | 3227 | 34°32′48″ N | 102°25′44″ E | -5.19 | 50.00 |
| NML2 | 3306 | 34°43′56″ N | 102°31′35″ E | -6.57 | 50.67 |
| AZ | 3497 | 33°40′14″ N | 101°52′02″ E | -12.73 | 53.58 |
| GH | 3508 | 34°13′22″ N | 102°15′57″ E | -13.19 | 55.58 |
| AWC1 | 3577 | 33°48′13″ N | 101°49′56″ E | -9.68 | 55.08 |
| MQ1 | 3580 | 33°48′02″ N | 101°48′55″ E | -9.44 | 55.25 |
| MQ2 | 3602 | 33°48′40″ N | 101°49′58″ E | -12.31 | 55.75 |
| AWC2 | 3634 | 33°51′42″ N | 101°53′50″ E | -14.35 | 56.42 |
| AWC3 | 3741 | 33°44′53″ N | 101°51′37″ E | -6.53 | 55.58 |
