# Supplementary material for: Selection by Pollinators on Floral Traits in Generalized Trollius ranunculoides (Ranunculaceae) along Altitudinal Gradients
Source: PLoS One. 2015 Feb 18;10(2):e0118299. doi: 10.1371/journal.pone.0118299 (PMC4334720; doi:10.1371/journal.pone.0118299)
Supplement: S2 Table — (DOCX) [file pone.0118299.s005.docx]

**Table S2. Number of observed visitors by functional groups and major species in 12 populations of *T. ranunculoides* along an altitudinal gradient.** (The visitors except for honey bees that occur only in HZ population, were classified into four functional groups: solitary bees, hoverflies, flies and ants; and the major species of each functional group are given).

|  | Populations | | | | | | | | | | | | |  |
| --- | --- | --- | --- | --- | --- | --- | --- | --- | --- | --- | --- | --- | --- | --- |
| Visitors | | HZ | NML1 | LQ1 | LQ2 | NML2 | AZ | GH | AWC1 | MQ1 | MQ2 | AWC2 | AWC3 | |
| Honey bees (*Apis cerana*) | | 195 | 0 | 0 | 0 | 0 | 0 | 0 | 0 | 0 | 0 | 0 | 0 | |
| **Solitary bees** | | 550 | 184 | 51 | 295 | 6 | 24 | 16 | 4 | 8 | 6 | 3 | 1 | |
| *Halictus sp* | | 230 | 105 | 23 | 149 | 4 | 10 | 6 | 3 | 4 | 2 |  |  | |
| *Lasioglossum sp* | | 259 | 56 | 28 | 124 | 1 | 10 | 8 | 1 | 3 | 3 | 3 | 1 | |
| *Melitta sp* | | 49 | 14 | 4 | 15 | 1 | 4 | 2 |  | 4 | 1 |  |  | |
| Other solitary bees | | 12 | 9 | 6 | 7 |  |  |  |  |  |  |  |  | |
| **Hoverflies** | | 143 | 26 | 43 | 10 | 0 | 52 | 26 | 0 | 0 | 0 | 11 | 0 | |
| *Episyrphus balteatus* | | 20 | 5 | 15 | 3 |  | 21 | 8 |  |  |  | 1 |  | |
| *Syrphus ribesii* | | 52 | 11 | 8 | 5 |  | 18 | 6 |  |  |  | 4 |  | |
| *Eristalis sp* | | 64 | 8 | 16 | 2 |  | 10 | 11 |  |  |  | 6 |  | |
| *Sphaerophoria sp* | | 4 | 2 | 2 |  |  |  |  |  |  |  |  |  | |
| Other hoverflies | | 3 |  | 2 |  |  | 3 | 1 |  |  |  |  |  | |
| **Flies** | | 287 | 235 | 182 | 230 | 191 | 1197 | 673 | 167 | 132 | 158 | 376 | 151 | |
| *Helina sp* | | 115 | 116 | 56 | 81 | 66 | 487 | 226 | 68 | 47 | 54 | 154 | 66 | |
| *Lucilia sp* | | 36 | 11 | 27 | 22 | 18 | 35 | 28 | 17 | 11 | 18 | 23 | 9 | |
| *Anthomyiidae sp* | | 103 | 88 | 71 | 101 | 95 | 562 | 284 | 54 | 66 | 57 | 139 | 62 | |
| Other flies | | 33 | 20 | 28 | 26 | 12 | 113 | 135 | 28 | 8 | 29 | 60 | 14 | |
| **Ants** (*Formica sp*) | | 9 | 0 | 8 | 46 | 15 | 122 | 3 | 656 | 94 | 7 | 13 | 488 | |
| **Others visitors** | | 45 | 10 | 2 | 10 | 3 | 7 | 5 | 31 | 4 | 22 | 4 | 4 | |
| Total visitors | | 1229 | 455 | 291 | 591 | 215 | 1402 | 723 | 858 | 238 | 193 | 407 | 644 | |
