# Supplementary material for: Selection by Pollinators on Floral Traits in Generalized Trollius ranunculoides (Ranunculaceae) along Altitudinal Gradients
Source: PLoS One. 2015 Feb 18;10(2):e0118299. doi: 10.1371/journal.pone.0118299 (PMC4334720; doi:10.1371/journal.pone.0118299)
Supplement: S3 Table — (DOCX) [file pone.0118299.s006.docx]

**Table S3. Loadings of floral traits of *T. ranunculoides* on the first four components (PCs) produced by a principle components analysis with a varimax rotation.**

|  | Loading | | | |
| --- | --- | --- | --- | --- |
| Flower trait (mm) | PC1 | PC2 | PC3 | PC4 |
| Sepal length | 0.723 | 0.479 | 0.273 | 0.16 |
| Sepal width | 0.894 | 0.258 | 0.17 | 0.212 |
| Petal length | 0.393 | 0.875 | 0.216 | 0.129 |
| Petal width | 0.194 | 0.111 | -0.026 | 0.974 |
| Flower height | 0.215 | 0.19 | 0.957 | -0.031 |
| Percentage variance explained (%) | 31.23 | 22.19 | 21.33 | 20.75 |
