# Supplementary material for: Selection by Pollinators on Floral Traits in Generalized Trollius ranunculoides (Ranunculaceae) along Altitudinal Gradients
Source: PLoS One. 2015 Feb 18;10(2):e0118299. doi: 10.1371/journal.pone.0118299 (PMC4334720; doi:10.1371/journal.pone.0118299)
Supplement: S4 Table — (DOCX) [file pone.0118299.s007.docx]

**Table S4. Selection differential (*S*), with fitness estimated as the visitation rate of all pollinators on the floral traits of 12 *T. ranunculoides* populations.**

| Fitness component | Population | Altitude (m) | Sepal  length | SE | *P*-value | Sepal  width | SE | *P*-value | Petal  length | SE | *P*-value | Petal  width | SE | *P*-value | Flower height | SE | *P*-value |
| --- | --- | --- | --- | --- | --- | --- | --- | --- | --- | --- | --- | --- | --- | --- | --- | --- | --- |
| Visitation rate | HZ | 2920 | 0.034 | 0.037 | 0.535 | **0.180** | **0.035** | **0.008** | **0.171** | **0.036** | **0.021** | **0.188** | **0.035** | **0.008** | -0.097 | 0.036 | 0.092 |
|  | NML1 | 3086 | 0.105 | 0.041 | 0.130 | **0.134** | **0.040** | **0.045** | 0.018 | 0.042 | 0.749 | 0.109 | 0.041 | 0.146 | 0.045 | 0.041 | 0.335 |
|  | LQ1 | 3180 | 0.108 | 0.057 | 0.176 | 0.076 | 0.057 | 0.437 | **0.192** | **0.056** | **0.026** | **0.156** | **0.056** | **0.048** | 0.048 | 0.057 | 0.624 |
|  | LQ2 | 3227 | 0.041 | 0.055 | 0.451 | 0.071 | 0.054 | 0.194 | 0.023 | 0.055 | 0.68 | -0.007 | 0.055 | 0.896 | **0.111** | **0.053** | **0.040** |
|  | NML2 | 3306 | 0.057 | 0.064 | 0.762 | **0.281** | **0.063** | **0.003** | 0.109 | 0.064 | 0.617 | 0.101 | 0.108 | 0.352 | -0.179 | 0.064 | 0.380 |
|  | AZ | 3497 | **0.093** | **0.047** | **0.050** | **0.084** | **0.047** | **0.080** | 0.070 | 0.047 | 0.142 | 0.029 | 0.048 | 0.548 | 0.076 | 0.047 | 0.113 |
|  | GH | 3508 | **0.204** | **0.071** | **0.006** | **0.263** | **0.068** | **0.001** | **0.220** | **0.071** | **0.003** | **0.210** | **0.071** | **0.005** | 0.045 | 0.076 | 0.554 |
|  | AWC1 | 3577 | **0.199** | **0.071** | **0.007** | 0.115 | 0.075 | 0.13 | 0.118 | 0.075 | 0.12 | 0.108 | 0.075 | 0.156 | 0.107 | 0.075 | 0.159 |
|  | MQ1 | 3580 | **0.224** | **0.069** | **0.021** | 0.174 | 0.070 | 0.288 | 0.073 | 0.071 | 0.508 | 0.104 | 0.071 | 0.411 | 0.027 | 0.072 | 0.703 |
|  | MQ2 | 3602 | 0.111 | 0.121 | 0.365 | **0.255** | **0.117** | **0.033** | 0.146 | 0.121 | 0.234 | 0.066 | 0.122 | 0.592 | **0.241** | **0.118** | **0.047** |
|  | AWC2 | 3634 | **0.158** | **0.069** | **0.028** | 0.135 | 0.071 | 0.066 | 0.062 | 0.08 | 0.443 | 0.128 | 0.068 | 0.066 | -0.043 | 0.072 | 0.554 |
|  | AWC3 | 3741 | **0.236** | **0.088** | **0.010** | **0.365** | **0.080** | **0.001** | **0.360** | **0.081** | **0.001** | **0.254** | **0.087** | **0.005** | **0.174** | **0.091** | **0.059** |
| Seed# per plant | HZ | 2920 | 0.026 | 0.045 | 0.560 | **0.105** | **0.043** | **0.017** | **0.093** | **0.044** | **0.037** | **0.125** | **0.042** | **0.004** | 0.040 | 0.048 | 0.408 |
|  | NML1 | 3086 |  |  |  |  |  |  |  |  |  |  |  |  |  |  |  |
|  | LQ1 | 3180 | 0.076 | 0.078 | 0.337 | 0.106 | 0.077 | 0.178 | -0.003 | 0.071 | 0.968 | 0.042 | 0.089 | 0.641 | 0.116 | 0.075 | 0.131 |
|  | LQ2 | 3227 | 0.114 | 0.093 | 0.226 | **0.279** | **0.087** | **0.002** | 0.163 | 0.092 | 0.084 | 0.028 | 0.094 | 0.772 | **0.319** | **0.084** | **0.0004** |
|  | NML2 | 3306 |  |  |  |  |  |  |  |  |  |  |  |  |  |  |  |
|  | AZ | 3497 | **0.152** | **0.062** | **0.019** | **0.156** | **0.055** | **0.007** | 0.080 | 0.067 | 0.237 | 0.061 | 0.059 | 0.311 | 0.104 | 0.056 | 0.072 |
|  | GH | 3508 | **0.126** | **0.058** | **0.034** | **0.154** | **0.060** | **0.014** | 0.108 | 0.057 | 0.064 | 0.016 | 0.062 | 0.798 | -0.011 | 0.061 | 0.857 |
|  | AWC1 | 3577 |  |  |  |  |  |  |  |  |  |  |  |  |  |  |  |
|  | MQ1 | 3580 | 0.029 | 0.066 | 0.666 | 0.030 | 0.066 | 0.652 | 0.114 | 0.064 | 0.081 | 0.055 | 0.066 | 0.411 | 0.068 | 0.066 | 0.302 |
|  | MQ2 | 3602 | -0.152 | 0.165 | 0.359 | 0.163 | 0.164 | 0.323 | -0.103 | 0.165 | 0.534 | 0.059 | 0.163 | 0.718 | 0.216 | 0.165 | 0.197 |
|  | AWC2 | 3634 |  |  |  |  |  |  |  |  |  |  |  |  |  |  |  |
|  | AWC3 | 3741 | 0.089 | 0.069 | 0.199 | **0.149** | **0.071** | **0.041** | 0.012 | 0.071 | 0.869 | 0.088 | 0.171 | 0.215 | **0.164** | **0.065** | **0.014** |
