# Supplementary material for: Selection by Pollinators on Floral Traits in Generalized Trollius ranunculoides (Ranunculaceae) along Altitudinal Gradients
Source: PLoS One. 2015 Feb 18;10(2):e0118299. doi: 10.1371/journal.pone.0118299 (PMC4334720; doi:10.1371/journal.pone.0118299)
Supplement: S5 Table — (DOCX) [file pone.0118299.s008.docx]

**Table S5.Selection gradients (β), with fitness estimated as the visitation rate of all pollinators on the loadings of these traits on the first four components (PCs) produced by a principal components analysis with a varimax rotation of 12 *T. ranunculoides* populations.**

|  | Population | Altitude (m) | PC1 | SE | *P-*value | PC2 | SE | *P*-value | PC3 | SE | *P*-value | PC4 | SE | *P*-value |
| --- | --- | --- | --- | --- | --- | --- | --- | --- | --- | --- | --- | --- | --- | --- |
| Visitation rate | HZ | 2920 | 0.090 | 0.037 | 0.164 | 0.091 | 0.038 | 0.269 | -0.120 | 0.038 | 0.057 | **0.174** | **0.035** | **0.013** |
|  | NML1 | 3086 | 0.113 | 0.043 | 0.138 | 0.030 | 0.042 | 0.511 | -0.007 | 0.044 | 0.798 | 0.074 | 0.044 | 0.402 |
|  | LQ1 | 3180 | 0.006 | 0.058 | 0.729 | 0.000 | 0.061 | 0.296 | -0.067 | 0.061 | 0.623 | **0.227** | **0.064** | **0.045** |
|  | LQ2 | 3227 | 0.083 | 0.057 | 0.152 | 0.010 | 0.059 | 0.870 | 0.108 | 0.058 | 0.070 | -0.044 | 0.055 | 0.425 |
|  | NML2 | 3306 | 0.107 | 0.066 | 0.390 | -0.027 | 0.072 | 0.522 | **-0.235** | **0.070** | **0.025** | 0.036 | 0.064 | 0.709 |
|  | AZ | 3497 | **0.122** | **0.058** | **0.041** | 0.083 | 0.060 | 0.169 | 0.058 | 0.051 | 0.259 | -0.004 | 0.053 | 0.936 |
|  | GH | 3508 | **0.163** | **0.076** | **0.035** | 0.085 | 0.076 | 0.265 | 0.013 | 0.076 | 0.870 | **0.157** | **0.076** | **0.044** |
|  | AWC1 | 3577 | **0.157** | **0.080** | **0.051** | 0.160 | 0.087 | 0.070 | 0.102 | 0.083 | 0.228 | 0.086 | 0.077 | 0.266 |
|  | MQ1 | 3580 | **0.208** | **0.074** | **0.046** | 0.045 | 0.082 | 0.740 | 0.011 | 0.079 | 0.668 | 0.061 | 0.073 | 0.644 |
|  | MQ2 | 3602 | 0.157 | 0.101 | 0.238 | -0.030 | 0.126 | 0.815 | -0.258 | 0.141 | 0.073 | 0.032 | 0.116 | 0.783 |
|  | AWC2 | 3634 | **0.175** | **0.083** | **0.041** | 0.013 | 0.076 | 0.867 | -0.018 | 0.081 | 0.828 | 0.131 | 0.074 | 0.084 |
|  | AWC3 | 3741 | **0.242** | **0.083** | **0.005** | **0.238** | **0.085** | **0.007** | 0.046 | 0.084 | 0.588 | 0.143 | 0.086 | 0.101 |
| Seed# per plant | HZ | 2920 | 0.069 | 0.047 | 0.149 | 0.058 | 0.047 | 0.219 | 0.039 | 0.051 | 0.454 | **0.113** | **0.043** | **0.010** |
|  | NML1 | 3086 |  |  |  |  |  |  |  |  |  |  |  |  |
|  | LQ1 | 3180 | 0.089 | 0.080 | 0.277 | 0.074 | 0.096 | 0.448 | 0.119 | 0.087 | 0.181 | -0.067 | 0.110 | 0.546 |
|  | LQ2 | 3227 | **0.284** | **0.089** | **0.002** | 0.088 | 0.093 | 0.347 | **0.289** | **0.091** | **0.003** | -0.085 | 0.087 | 0.330 |
|  | NML2 | 3306 |  |  |  |  |  |  |  |  |  |  |  |  |
|  | AZ | 3497 | **0.206** | **0.068** | **0.005** | 0.108 | 0.077 | 0.167 | 0.089 | 0.055 | 0.116 | 0.006 | 0.060 | 0.919 |
|  | GH | 3508 | **0.180** | **0.063** | **0.007** | 0.044 | 0.060 | 0.469 | -0.006 | 0.062 | 0.919 | -0.049 | 0.061 | 0.421 |
|  | AWC1 | 3577 |  |  |  |  |  |  |  |  |  |  |  |  |
|  | MQ1 | 3580 | -0.009 | 0.067 | 0.890 | 0.135 | 0.074 | 0.074 | 0.097 | 0.071 | 0.180 | 0.032 | 0.066 | 0.632 |
|  | MQ2 | 3602 | 0.259 | 0.182 | 0.162 | -0.126 | 0.170 | 0.463 | **0.419** | **0.190** | **0.033** | 0.048 | 0.156 | 0.758 |
|  | AWC2 | 3634 |  |  |  |  |  |  |  |  |  |  |  |  |
|  | AWC3 | 3741 | 0.106 | 0.068 | 0.123 | -0.061 | 0.067 | 0.367 | **0.134** | **0.065** | **0.044** | 0.064 | 0.069 | 0.357 |
