# Supplementary material for: Selection by Pollinators on Floral Traits in Generalized Trollius ranunculoides (Ranunculaceae) along Altitudinal Gradients
Source: PLoS One. 2015 Feb 18;10(2):e0118299. doi: 10.1371/journal.pone.0118299 (PMC4334720; doi:10.1371/journal.pone.0118299)
Supplement: S6 Table — (DOCX) [file pone.0118299.s009.docx]

**Table S6.** The relationships between visitation rate of pollinators and seed set for 11 populations of *T. ranunculoides.*

| Population | Altitude (m) | N | *r* | *P* |
| --- | --- | --- | --- | --- |
| HZ | 2920 | 70 | -0.002 | 0.988 |
| NML1 | 3086 | 24 | **0.443** | **0.03** |
| LQ1 | 3180 | 35 | -0.076 | 0.66 |
| LQ2 | 3227 | 57 | **0.292** | **0.026** |
| NML2 | 3306 | 16 | -0.017 | 0.948 |
| AZ | 3497 | 40 | **0.349** | **0.05** |
| GH | 3508 | 51 | -0.028 | 0.843 |
| AWC1 | 3577 | 14 | -0.187 | 0.522 |
| MQ1 | 3580 | 49 | -0.121 | 0.402 |
| MQ2 | 3602 | 47 | 0.037 | 0.801 |
| AWC2 | 3634 | - | - | - |
| AWC3 | 3741 | 53 | -0.137 | 0.324 |
